# The Impact of Mortality Salience on Purchase Intention and Creativity Evaluation on Products During COVID-19 Pandemic

**DOI:** 10.3389/fpsyg.2021.601383

**Published:** 2021-12-20

**Authors:** Chong Zu, Xiang Zhou, Yu-Xin Cui, Yan-Fang Liu, Yue-Xin Hu, Dong-Qi Li, Hui Zeng

**Affiliations:** ^1^Department of Social Psychology, Nankai University, Tianjin, China; ^2^School of Economics, Nankai University, Tianjin, China

**Keywords:** COVID-19, mortality salience, purchase intention, creativity, cultural worldview defense

## Abstract

In the environment of COVID-19, people are faced with mortality salience (MS) and socioeconomic crisis. According to the terror management theory, the MS would lead to particular consumption attitudes and behaviors caused by the self-esteem and cultural worldview defense. The creativity as a potential value of products needs to be examined to explore how the MS changed the creativity evaluation of three types of products categorized into normal, renovative, and innovative products, based on the degree of originality (Zhang et al., 2019). Two experiments were conducted to examine (1) the MS effect on the creativity and purchase intention evaluation and (2) both MS and country-of-origin effect on the evaluations. The results show that usefulness and purchase intention are affected by both effects, and the novelty is mainly affected by MS.

## Introduction

In the COVID-19 pandemic, people face the risk of infection, the pain of illness, and the threat of death anytime. Under these circumstances, daily news, social networks, daily necessities related to the epidemic, and social distance remind people of the death they may encounter (Menzies and Menzies, 2020). Besides, people confront the socioeconomic crisis (Nicola et al., 2020). The cognitive processes produced by mortality salience (MS) are the motives of people’s behaviors, which is a director of social psychology interpretation from terror management theory (TMT) (Solomon et al., 1991). According to TMT, our awareness of death would trigger our anxiety (Greenberg et al., 2003; Zhou, 2020) and thereby perform a series of actions to reinforce a sense of meaning, including cultural worldviews defense and self-esteem efforts (Greenberg et al., 1986; Ferraro et al., 2005). Thus, people who lack meaning in life would be more prone to lead to death anxiety and activate terror management after MS manipulation (Routledge and Juhl, 2010). Thereby, they would positively evaluate the in-group members and more negatively on out-groupers (Greenberg et al., 1990) to achieve a sense of meaning in life. People attempt to gain an insight into symbolic immortality through a meaningful life and bolster self-esteem through aligning well with the value prescribed by the worldview (Elad-Strenger, 2016).

In other words, MS can be considered as the cause of some kinds of behaviors. Thereinto, consumption behaviors that are affected by the awareness of mortality can be observed easily, which can be explained through TMT, which leads to materialism consumption (Kasser and Sheldon, 2000) and domestic product preference (Friese and Hofmann, 2008). These consumption behavior tendencies are affected by product evaluation and perceived product value (Hui and Zhou, 2002). Moreover, these attitude tendencies of products are related to the values of consumers who tend to deal with the death anxiety through the purchase of valuable products or high evaluation of such products, which would reflect the worth of consumers (Audrin et al., 2018). In general terms, according to the studies of consumption behavior, the brand and function of products that can reflect the value of products are mainly considered as the aspects of products’ values (Fransen et al., 2008; McCabe and Bartholow, 2019). In addition to these, the creativity of products can also be regarded as a potential value to improve the purchase intention of people (Horn and Salvendy, 2006).

Creativity plays a contradictory role in the cognitive processes after MS that influences the behaviors of people. On the one hand, radical innovation inconsistent with social consensus may decrease the evaluation of creative achievements (Boeuf, 2019). On the other hand, once the social consensus recognizes the creative accomplishments, they would become a new part of social culture and a valuable heritage worth preserving, encouraging people to improve the evaluation of these achievements on creativity (Arndt et al., 1999). Whether the social consensus can accept it may be related to the degree of products’ originality because radical innovation would have adverse effects on consumers’ evaluation (Mugge and Dahl, 2013). Zhang et al. (2019) suggested a category method based on the originality of products: products can be categorized into normal products (i.e., prototype products), renovative products (the products that create based on the prototype products and improved in structure and function with a similar appearance to prototype ones), and innovative products (the products that do not rely on any existing products and can meet specific unmet needs of human beings). They also found that the novelty of products is more uncertain than usefulness as an evaluation dimension, making it easier for people to conform. Similarly, Yang et al. (2020) also found that the self-uncertainty caused by MS would push people into conformity.

The MS can activate defense mechanisms, including cultural worldview defense and self-esteem, to protect people from death anxiety (Solomon et al., 1991). The TMT holds the view that after MS, people tend to improve the preference of internal groups and the exclusion of external groups, which is manifested as cultural worldview defense. In the consumption studies, the country-of-origin (COO) effect is similar to such cultural worldview defense (Maheswaran and Agrawal, 2004) because MS would lead to ethnocentrism, measured through the COO effect. COO is an aspect of product information that would affect the evaluation of the products by consumers as a cognitive and cultural cue. Such an effect of the evaluation and purchase preference to the domestic products is called the COO effect (Verlegh and Steenkamp, 1999). Hui and Zhou (2002) investigated the influence of COO on product value perception and purchase intention. They found that COO directly affected the product evaluation and an indirect effect on perceived product value that would determine purchase intention, which is also affected by brand name and price factors. Besides, Friese and Hofmann (2008) examined the consumption of food products and found that participants prefer domestic products after MS. Also, reminders of death would prompt participants to purchase domestic products for a sense of control (Liu et al., 2014). Such preference for domestic products is related to their quality (Hui and Zhou, 2002) and regulated by the esteem of individuals and individualism. Taking a study conducted in the developing country as an example, people prefer to give a high evaluation of imported products to enhance their self-esteem (Cutright et al., 2011; Banerjee et al., 2019). As a whole, the study of the COO can examine the influence of cultural worldview defense on the purchase intention of people and the evaluation of products (Maheswaran and Agrawal, 2004).

Therefore, the pandemic environment reminds people of death anytime, through infection data, mortality reported publicly, and any information about COVID-19. Not only would death anxiety change the behaviors of people but also the socioeconomic crisis caused by such severe conditions leads to a sense of fear and negative emotion. However, the effects of MS are at the cognitive level that would change the behaviors of people fundamentally, so that the investigation of the MS effect is necessary. The TMT can explain the behaviors of people caused by MS from the needs of self-esteem and the consequent cultural worldview defense. After MS, people have a sense of insecurity and anxiety. Thus, people need to evaluate positively and affirm their value to alleviate these negative cognition states, which are manifested in need of self-esteem and the cultural worldview defense (Arndt et al., 1997). The consumption contains both cognition and behavior aspects that occur and affect the lives of people ubiquitously and is influenced by MS significantly. Nevertheless, present researches mainly focus on the functionality (e.g., food) or value (e.g., brand) of the products (Maheswaran and Agrawal, 2004; McCabe and Bartholow, 2019). The creativity evaluation, as an important factor in both individual and team researches, need to be taken seriously (Zhou et al., 2019; Zu et al., 2019). In the face of more and more creative products, the impact of MS on the creativity evaluation of products in various degrees of originality is worth studying. In general, creativity can be evaluated through usefulness and novelty (Diedrich et al., 2015). Thus, this study adopts the categorization method of products by Zhang et al. (2019) based on originality, as well as the experimental materials (i.e., product images), to explore the influence of MS and the subsequent improvement of cultural worldview defense (manifested as COO effect) on the creativity evaluation of the products (usefulness and novelty) and purchase intention.

The study conducted two experiments to explore the MS effect on creativity evaluation and purchase intention under the environment of COVID-19. Experiment 1 examined the impact of MS on the evaluation of three types of products, which are categorized into normal, renovative, and innovative products, on usefulness, novelty, and purchase intention. Experiment 2 examined the COO effect after MS on the same dimensions of evaluation of the same products. We suppose that people would evaluate normal products more positively after MS and be more likely affected by the COO effect. In contrast, creative products and their novelty evaluation would be mainly affected by MS.

## Experiment 1

In both experiments, participants are asked to evaluate three types of products (normal, renovative, and innovative products) on their usefulness, novelty, and purchase intention of participants. In experiment 1, we examined the variations in three dimensions of the three types of products, before and after MS and tourist industry crisis manipulation.

### Method

#### Participants

Participants were recruited online from various regions of China in both experiments that were conducted using online questionnaires to manipulate and measure. The answering time was limited, and the invalid question was designed (“Please select the number 6”) to ensure the completion of participants and accuracy of the experiment. In experiment 1, 41 participants were recruited through social networks (*M*_*age*_ = 21.83; 68.3% of women) and randomly assigned to the experimental group (MS manipulation) and the control group (tourist industry crisis manipulation). The type of products is the within-subject variable, and participants from the two groups were asked to evaluate the usefulness, novelty, and purchase intention of three types of products, before and after two different manipulations.

#### Materials and Procedures

First, two groups were asked to complete the pre-test of three types of products (normal, renovative, and innovative). The study adopted the same product images and categorization method, and the definitions of renovative products and innovative products used by Zhang et al. (2019). Besides, for the sake of the accuracy of product images, the study adopted the same criteria of categorization: renovative and innovative products were significantly different in answer to the question “whether the product was created based on a prototype” (*t* = −2.74, *p* < 0.01) (Zhang et al., 2019). The product images were designed by experts with a black background and only depicted the product itself, below which was a sentence to inform participants of the product function in the simple SVO structure (Zhang et al., 2019). According to the categorization method, 10 products in each category (30 in total) included kitchen utensils, clothing, furniture, and electronic products to balance the product function.

In the beginning, all participants were asked to evaluate three types of products: usefulness, novelty, and purchase intention with a 7-point scale (1, very low; 7, very high). According to the internal consistency test results, the usefulness, novelty, and purchase intention variables of the three types of products had acceptable Cronbach’s α coefficients above 0.70, and most were above 0.80 (see Table 1).

**TABLE 1:** In Experiment 1, Cronbach’s α coefficients of usefulness, novelty, and purchase intention variables of three types of products.

	Normal (*n* = 10)	Innovative (*n* = 10)	Renovative (*n* = 10)
Usefulness	0.904	0.772	0.884
Novelty	0.955	0.756	0.734
Purchase Intention	0.915	0.842	0.894

Then, participants were randomly assigned to the experimental group and the control group. In the experimental group, participants were asked to read a text describing infection, death rate, and the pandemic severity, whereas, in the control group, participants were asked to read a text describing the tourist industry crisis and severe unemployment under the pandemic situation. Having finished reading the text, participants were asked to answer an open-ended question, “How do you feel about the death (in the control group: tourist industry crisis) under the COVID-19,” and then to write down their answers. Based on the above-mentioned manipulations, participants then completed a positive and negative emotion scale (PANAS; Watson and Clark, 1991) to test the sense of fear and a delay task.

Eventually, as a post-time test, participants evaluated the usefulness, novelty, and purchase intention of three types of products (30 in total) once again. The variations of evaluation before and after MS were analyzed through paired *t*-test and examined the MS effect and the influence of product category through ANOVA.

### Mortality Salience

According to previous answers that participants wrote to express their feelings of death, if participants wrote words such as “恐惧” (fear), “死” (death), and other relative answers, the responses were coded as 1. Through this method, 78.9% of participants depicted their anxiety and fear of death caused by COVID-19 through the mentioned coded words. In the control group, the manipulation text was about tourist industry crisis to remind participants of the socioeconomic crisis caused by the pandemic situation, and if the answers of participants included words such as “经济危机” (economic crisis), “失业” (unemployment), and other relative words, the responses were coded as 2. Through this method, 68.2% of participants reported their worries about the economic situation using the abovementioned coded words.

The result of the *t*-test on the item of fear in the PANAS showed an insignificant difference between the two groups (*p* = 0.188), meaning that the changes of evaluation scores after manipulations were not caused by emotion but by the MS.

### Analyses and Results

Experiment 1 was a 2 (manipulation: mortality vs. tourist industry crisis) × 3 (product category: normal vs. innovative vs. renovative products) mixed design. A paired *t*-test was conducted to examine the variations in evaluations before and after MS within the experimental group. A 2 × 3 ANOVA was then conducted to examine the differences in the evaluation scores between pre-test and post-test (△*M* = *M*_*post*_ − *M*_*pre*_) to see if they were affected by two different manipulations or the product category.

#### *T*-Test

First, a paired *t*-test was conducted within the experimental group. Compared with the evaluation scores before the operationalization, the results showed that after MS, the differences between usefulness and novelty were insignificant. However, in terms of normal products, the purchase intention was increased after MS [*t*_(18)_ = −2.43, *p* = 0.026, *d* = −0.56].

#### Analyses of Variance

The impact of MS on product evaluation results needs to be examined by comparing the two groups. Because of the subjectivity of product evaluation, the dependent variables used in the ANOVA were the differences in evaluation scores between pre-test and post-test (△*M* = *M*_*post*_ − *M*_*pre*_). Even if there were no significant differences in the paired *t*-test, the effect of MS and product category might be significant compared with △M. Before the ANOVA, the kurtosis and skewness, as well as the *W*-test on dependent variables (△M) used in the analyses, were measured (see Table 2). In general, most *p* values are above 0.05 in the *W*-test, which means the dependent variables are normally distributed.

**TABLE 2:** In Experiment 1, the measurements of kurtosis and skewness as well as the *W*-test on the dependent variables (the differences in the evaluation scores between pre-test and post-test, △*M* = *M*_*post*_ − *M*_*pre*_).

	Normal			Innovative			Renovative		
	Skewness	Kurtosis	Shapiro-Wilk	Skewness	Kurtosis	Shapiro-Wilk	Skewness	Kurtosis	Shapiro-Wilk
Usefulness	1.066	0.994	0.916 (*p* = 0.096)	–0.929	1.686	0.943 (*p* = 0.282)	0.498	0.086	0.961 (*p* = 0.597)
Novelty	0.681	–0.591	0.904 (*p* = 0.058)	0.460	0.124	0.963 (*p* = 0.638)	0.346	–0.575	0.963 (*p* = 0.623)
Purchase Intention	0.699	–0.449	0.933 (*p* = 0.194)	0.284	–0.306	0.951 (*p* = 0.407)	0.600	0.157	0.942 (*p* = 0.285)

**FIGURE 1:**
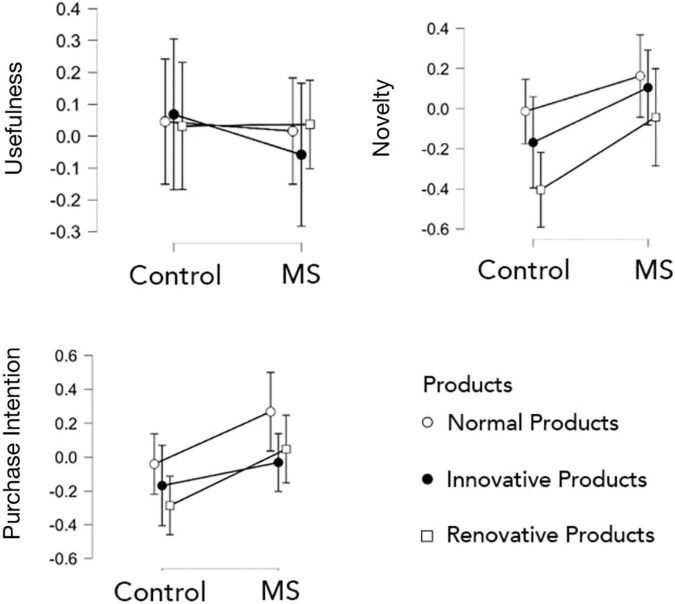
The ANONA analysis results of Experiment 1.

We analyzed differences in the evaluation scores (△M of usefulness, novelty, and purchase intention) *via* 2 (manipulation) × 3 (product category) analyses of variance (ANOVA) (see Table 3 and Figure 1). First, it could be found that there was no interaction effect between manipulation and product category on the three dependent variables.

**TABLE 3:** The results of ANOVA of Experiment 1.

	Mean (SD)					
	Mortality salience (MS) (*n* = 19)			Control (*n* = 22)		
	Normal	Innovative	Renovative	Normal	Innovative	Renovative
Usefulness	0.016 (0.35)	–0.058 (0.47)	0.037 (0.29)	0.045 (0.44)	0.068 (0.53)	0.032 (0.45)
Novelty	0.163 (0.43)	0.105 (0.39)	–0.042 (0.50)	–0.014 (0.36)	–0.168 (0.51)	–0.405 (0.43)
Purchase Intention	0.268 (0.48)	–0.032 (0.35)	0.047 (0.42)	–0.041 (0.40)	–0.168 (0.54)	–0.286 (0.39)

In terms of usefulness, the main effects of manipulation and product category were all insignificant, consistent with the result of the *t*-test. It could be considered that MS did not affect the usefulness of three types of products.

In terms of novelty, the main effects of manipulation and product category were significant, *F*(1,39) = 7.947, *p* < 0.01, η*^2^* = 0.084; *F*(2,78) = 6.330, *p* < 0.01, η*^2^* = 0.070, respectively. According to the *post hoc* test results, when the results of different types of products were averaged, in general, the novelty scores increased after MS (*t* = −2.819, *p* < 0.01, *d* = −0.44). In the control group, the novelty scores of normal and innovative products decreased after the manipulation (△*M* = −0.014 and △*M* = −0.168, respectively), while in the experimental group the novelty scores improved (△*M* = 0.163 and △*M* = 0.105, respectively). Besides, the main effect of product category on the novelty mainly occurred between normal and renovative products: the variation of the novelty of normal products before and after manipulation was significantly higher than that of renovative products (*t* = 3.510, *p* < 0.01, *d* = 0.55).

In terms of purchase intention, two main effects of manipulation and product category were significant, *F*(1,39) = 7.085, *p* < 0.011, η*^2^* = 0.080; *F*(2,78) = 4.919, *p* = 0.010, η*^2^* = 0.053, respectively. After manipulation of the MS, the purchase intention scores increased (*t* = −2.662, *p* = 0.010, *d* = −0.42), especially on normal products (control group: △*M* = −0.041; experimental group: △*M* = 0.268).

Both within-group *t*-test and ANOVA results showed that the purchase intention of normal products increased significantly after manipulation of the MS compared with the control group. The novelty was significantly affected by MS, showing significant improvements in the novelty of three types of products after MS. However, the usefulness was hardly affected by the effect of MS.

## Experiment 2

In Experiment 2, we examined the COO effect after MS manipulation in the experimental group and after tourist industry crisis manipulation in the control group. After the manipulation, participants were told about the COO of products that were domestic or imported. Then, the study examined the dependent variables (the differences in the evaluation scores between pre-test and post-test, △*M* = *M*_*post*_ − *M*_*pre*_) between the effects of manipulation and COO of products through ANOVA. In Experiment 2, the products were still categorized into normal, innovative, and renovative products, but the variations of evaluations were discussed, respectively, from these three types of products.

### Method

#### Participants

Same as Experiment 1, 82 participants from different regions of China were recruited online and completed Experiment 2 (*M*_*age*_ = 22.24; 58.5% female), who were then randomly assigned into the experimental group (MS) and the control group (tourist industry crisis). The assignment of grouping was conducted at the beginning of the experiment. In Experiment 2, the study examined the variations of evaluations before and after knowing the COO of three types of products, after the manipulations. There was a hypothesis that after MS, the culture worldview defense would increase significantly. Therefore, the COO effect on the variations of evaluations was examined based on the increasing cultural worldview defense.

#### Materials and Procedures

First, participants were assigned into two groups. The manipulation method and experimental materials (manipulation text, questions, PANAS, and product images) were consistent with those in Experiment 1.

Participants were then asked to evaluate three types of products (10 in each category; 30 in total) on usefulness, novelty, and purchase intention with a 7-point scale (1, very low; 7, very high).

Following the first evaluation, the cultural worldview defense was measured using a Chinese text that depicted Chinese culture positively and negatively, and participants were asked to evaluate the view of authors with a 10-point scale (1, totally disagreed; 10, absolutely agreed).

Finally, as a post-test, participants evaluated the usefulness, novelty, and purchase intention of three types of products (30 in total) for a second time. In the second evaluation, the study randomly selected half of each category of products (five products) labeled as domestic products and half as imported products. Participants were told about the COO of products by a sentence below the product images that were the same as used in Experiment 1, which meant each category of products (*n* = 10) were randomly divided into half domestic (*n* = 5) and half imported (*n* = 5). According to the internal consistency test results, the usefulness, novelty, and purchase intention variables of the three types of products and two different countries of origin products had acceptable Cronbach’s α coefficients above 0.70 and most were above 0.80 (see Table 4).

**TABLE 4:** In Experiment 2, Cronbach’s α coefficients of usefulness, novelty, and purchase intention variables of three types of domestic and imported products.

	Normal (*n* = 10)		Innovative (*n* = 10)		Renovative (*n* = 10)	
	Domestic (*n* = 5)	Import (*n* = 5)	Domestic (*n* = 5)	Import (*n* = 5)	Domestic (*n* = 5)	Import (*n* = 5)
Usefulness	0.813	0.828	0.839	0.800	0.835	0.900
Novelty	0.929	0.948	0.764	0.815	0.806	0.852
Purchase Intention	0.893	0.843	0.910	0.855	0.895	0.905

### Mortality Salience

According to previous answers that participants wrote to express their feelings of death, if participants wrote words such as “恐惧” (fear), “死” (death), and other relative answers, the responses were coded as 1. Through this method, 84.2% of participants depicted their anxiety and fear of the death caused by COVID-19 through the mentioned coded words. In the control group, the manipulation text was about tourist industry crisis to remind participants of the socioeconomic crisis caused by the pandemic situation, and if participants wrote words such as “经济危机” (economic crisis), “失业” (unemployment), and other relative words, the responses were coded as 2. Through this method, 65.9% of participants reported their worries about the economic situation using the abovementioned coded words.

The result of the *t*-test on the item of fear in the PANAS showed an insignificant difference between the two groups (*p* = 0.692), meaning that the changes of evaluation scores after manipulations were not caused by emotion but by the MS.

### Analyses and Results

#### *T*-Test

First, an independent sample *t*-test of cultural worldview defense scores was conducted between the experimental group and the control group. The results showed that there was a significant difference between two manipulations [*t*_(80)_ = −2.070, *p* < 0.05, *d* = −0.46]. Compared with the control group (*M* = 79.11, *SD* = 15.99), after the effect of MS, the score of cultural worldview defense was increased (*M* = 87.37, *SD* = 20.10).

Second, a paired *t*-test was conducted within the experimental group. The results showed that in the MS manipulation group, after knowing the COO, the usefulness of all types of domestic products increased and imported products decreased (insignificant). Thereinto, only the usefulness of renovative domestic products significantly increased [*t*_(37)_ = −3.435, *p* < 0.001, *d* = −0.56], while the usefulness of innovative domestic products increased with marginal significance [*t*_(37)_ = −1.861, *p* = 0.071, *d* = −0.30]. Besides, compared with the pre-test that participants were not told about the COO, the purchase intention of renovative domestic products increased significantly [*t*_(37)_ = −1.993, *p* = 0.054, *d* = −0.32].

In the condition of MS, compared with pre-test, after participants were told about the COO, novelty, and purchase intention of normal domestic products increased significantly [novelty: *t*_(37)_ = −2.376, *p* = 0.023, *d* = −0.39; purchase intention: *t*_(37)_ = −3.177, *p* < 0.01, *d* = −0.52], while the purchase intention of normal imported products decreased insignificantly [*t*_(37)_ = 1.258, *p* = 0.216].

#### Analyses of Variance

Before the ANOVA, the kurtosis and skewness were measured, and the *W*-test on the dependent variables (△M) was used in the analyses (see Table 5). In general, most *p* values were above 0.05 in the *W*-test, which meant that the values of dependent variables were normally distributed. In Experiment 2, the results are discussed in three product categories, respectively. The experiment examined the effects of the two manipulations and two different COO on dependent variables (differences in the evaluation scores between pre-test and post-test, △*M* = *M*_*post*_ − *M*_*pre*_) in the ANOVA.

**TABLE 5:** In Experiment 2, the measurements of kurtosis and skewness as well as the *W*-test on the dependent variables (the differences in the evaluation scores between pre-test and post-test, △*M* = *M*_*post*_ − *M*_*pre*_).

	Normal			Innovative			Renovative		
	Skewness	Kurtosis	Shapiro-Wilk	Skewness	Kurtosis	Shapiro-Wilk	Skewness	Kurtosis	Shapiro-Wilk
Usefulness	0.344	–0.370	0.965 (*p* = 0.269)	0.663	–0.209	0.938 (*p* = 0.036)	0.261	–0.242	0.974 (*p* = 0.520)
Novelty	0.108	–0.446	0.974 (*p* = 0.496)	–0.023	0.489	0.982 (*p* = 0.782)	–0.022	0.368	0.982 (*p* = 0.790)
Purchase Intention	0.645	–0.484	0.924 (*p* = 0.013)	0.261	0.861	0.957 (*p* = 0.152)	0.247	0.485	0.974 (*p* = 0.501)

**FIGURE 2:**
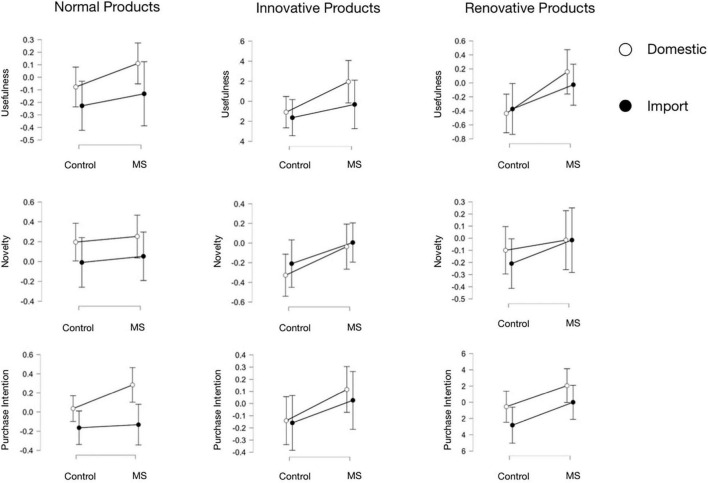
The ANONA analysis results of Experiment 2.

Experiment 2 was a 2 (manipulation: mortality vs. tourist industry crisis) × 2 (COO: domestic vs. imported) mixed design. The study analyzed the results of three product categories, respectively, *via* 2 × 2 ANOVA (see Table 6 and Figure 2). The dependent variables were different in evaluation scores between post-test and pre-test (△M). First, ANOVA results did not show a significant interaction effect of manipulation of COO of products. According to the result of the independent sample *t*-test, the manipulation of the COO of products was based on the cultural worldview defense in the MS group, which meant that the COO effect could be seen as the manifestation of the effect of cultural worldview defense.

**TABLE 6:** The results of ANOVA of Experiment 2.

		Mean (SD)			
		MS (*n* = 38)		Control (*n* = 44)	
		Domestic	Import	Domestic	Import
Normal	Usefulness	0.111 (0.50)	–0.132 (0.78)	–0.077 (0.52)	–0.227 (0.64)
	Novelty	0.253 (0.66)	0.053 (0.74)	0.195 (0.62)	–0.009 (0.82)
	Purchase Intention	0.284 (0.55)	–0.132 (0.65)	0.036 (0.44)	–0.164 (0.57)
Innovative	Usefulness	0.195 (0.65)	–0.032 (0.74)	–0.109 (0.52)	–0.164 (0.60)
	Novelty	–0.037 (0.70)	0.005 (0.61)	–0.327 (0.70)	–0.209 (0.80)
	Purchase Intention	0.116 (0.57)	0.026 (0.73)	–0.141 (0.65)	–0.159 (0.74)
Renovative	Usefulness	0.158 (0.96)	–0.026 (0.90)	–0.436 (0.91)	–0.373 (1.19)
	Novelty	–0.016 (0.74)	–0.016 (0.81)	–0.100 (0.64)	–0.209 (0.67)
	Purchase Intention	0.205 (0.64)	0.000 (0.64)	–0.055 (0.63)	–0.282 (0.73)

In terms of normal products, △Ms of usefulness, novelty, and purchase intention all had a significant main effect of COO, *F*(1,80) = 7.851, *p* < 0.01, η*^2^* = 0.025; *F*(1,80) = 6.589, *p* = 0.012, η*^2^* = 0.020; *F*(1,80) = 17.167, *p* < 0.001, η*^2^* = 0.071, respectively, and there was no significant main effect of MS. According to the results of the *post hoc* test, the usefulness of domestic products increased significantly (*t* = 2.802, *p* < 0.01, *d* = 0.31), compared with imported products, while the improvement of novelty of domestic products was significant (*t* = 2.567, *p* = 0.012, *d* = 0.28). The effect of COO was significant on purchase intention of normal products, which showed that purchase intention scores of domestic products increased compared with imports (*t* = 4.143, *p* < 0.001, *d* = 0.46), compared with scores of the imported. In Experiment 2, usefulness and purchase intention of normal domestic products increased, and these variations were influenced by the COO effect.

In terms of innovative products, three evaluation dimensions (usefulness, novelty, and purchase intention) had a margin significant main effect of MS, *F*(1,80) = 3.541, *p* = 0.063, η*^2^* = 0.030; *F*(1,80) = 3.431, *p* = 0.068, η*^2^* = 0.031; *F*(1,80) = 3.011, *p* = 0.087, η*^2^* = 0.026, respectively. In general, the COO did not affect the evaluations of participants, and the variations were mainly from the manipulation effect, while only the evaluation of usefulness would be affected by the notice of COO of the products, *F*(1,80) = 3.515, *p* = 0.064, η*^2^* = 0.012. After MS, usefulness scores increased significantly (*t* = −1.882, *p* = 0.063, *d* = −0.21), especially on domestic products (control group: △*M* = −0.109; MS group: △*M* = 0.195), while the novelty scores decreased in both groups after the notice of the COO of products. Compared with the control group, after the manipulation of MS, purchase intention scores increased significantly (*t* = −1.735, *p* = 0.087, *d* = −0.19). Thus, after the manipulation of MS, the usefulness and purchase intention of innovative domestic products increased, and there was no significant difference in novelty between different countries of origin.

In terms of renovative products, the usefulness had a significant main effect of MS and the main effect of COO, *F*(1,80) = 4.805, *p* = 0.031, η*^2^* = 0.028; *F*(1,80) = 8.331, *p* < 0.01, η*^2^* = 0.046, and purchase intention had significant main effects of MS and COO, *F*(1,80) = 5.321, *p* = 0.024, η*^2^* = 0.040; *F*(1,80) = 6.074, *p* = 0.016, η*^2^* = 0.025, respectively. The novelty had no significant main effect and interaction effect. According to the results of *post hoc* test, compared with the control group, usefulness of renovative products increased significantly after the manipulation of MS (*t* = −2.192, *p* = 0.031, *d* = −0.24), especially on domestic products (control group: △*M* = −0.436; MS group: △*M* = 0.158, *t* = −2.886, *p* < 0.01, *d* = −0.32). The purchase intention of renovative products was affected by the MS and COO effect: compared with the control group, purchase intention increased significantly (*t* = −2.307, *p* = 0.024, *d* = −0.26), after participants knowing that the products are domestic, the purchase intention increased significantly (*t* = 2.464, *p* = 0.016, *d* = 0.27). Thus, after the manipulation of MS, the usefulness and purchase intention of renovative domestic products increased.

Given that after the manipulation of MS, cultural worldview defense increased, and the post-test was conducted under this condition. According to the ANOVA results, evaluations of normal products were affected by the COO, which showed a preference for domestic products. The innovative and renovative products were mainly affected by MS, while novelty scores had insignificant differences between the domestic and the imported.

## Discussion and Conclusion

According to results in Experiment 1, the purchase intention of normal products significantly increased after MS. The novelty was also affected by the manipulation, manifested as an increasing variety of novelty after MS, compared with the control group. The usefulness was hardly affected by the manipulation. In Experiment 2, after the manipulation of MS, the usefulness and purchase intention of three types of products increased significantly. Normal products were mainly affected by cultural worldview defense (COO). In contrast, innovative and renovative products were affected by MS manipulation. In terms of novelty, normal products were of marginal significance on the main effect of COO. Although the novelty variation of innovative products was higher than the control group, domestic and import innovative products all decreased in the post-test. For the renovative products, the MS and COO effect does not affect the novelty.

The categories (innovative and renovative) and evaluation dimension (novelty) related to novelty and originality were mainly affected by the MS. In contrast, usefulness and purchase intention were primarily affected by cultural worldview defense (COO), based on the MS effect. These analyses indicate that creativity evaluation is affected directly by the cognitive influence of death awareness. In comparison, usefulness and purchase intention are affected by the COO as a cultural cue of the value of products.

Creativity, as an attribute reflecting the product value, would prompt people to improve the novelty evaluation of products due to the need for value pursing after the effect of MS, which is consistent with the result of Experiment 1. Besides, individuals are more creative when they need to leave a legacy under the effect of MS if creativity does accord with their social values (Sligte et al., 2013), which indicates the effects of death reminders on creativity. Accordingly, in the context of the salience of mortality, creative behaviors would interrupt the social connections of individuals, which would lead to guilty and weaken their cultural worldview defense (Arndt et al., 1999; Routledge et al., 2004). However, creativity can also enhance a sense of meaningfulness and well-being of life to alleviate death anxiety when people face death (Routledge and Arndt, 2009).

Meanwhile, the uncertainty caused by creativity would lead to conformity behaviors (Zhang et al., 2019), which means the creativity evaluation would be directly affected by the cognitive states of individuals. Yang et al. (2020) confirmed the mediation effect of self-uncertainty between MS and conformity. Besides, Experiment 1 showed that novelty scores increased after the manipulation of MS, and results in Experiment 2 indicated that evaluations of innovative and renovative products are affected by MS. These results show that MS leads people to evaluate creative products and their novelty more positively. Moreover, combining results in this study with previous researches, the uncertainty of novelty and originality can be seen as the mediator between these positive evaluations and MS. Therefore, future research can be conducted to examine the role of self-esteem between MS and the creative evaluation of products.

The manipulation of products’ COO divided products into their own cultural products (domestic) and products from other countries (imported), which would activate people’s cultural worldview defense. Higher defense scores mean a higher preference to own cultural products, which is consistent with the results of Experiment 2: the purchase intention of normal and renovative products increased after the manipulation of MS and was affected by the COO effect positively. The usefulness is only affected by the COO effect whose renovative and normal products are improved. The influences of the COO might be affected by the quality of products (Hui and Zhou, 2002). Nevertheless, the products of product images labeled domestic and imported were the same in the pre-test, which informs participants that there were no quality differences between domestic and imported products.

The above theoretical inference needs further certification through efforts of other studies to focus more on the cognitive processes that might influence the creativity evaluation for a more accurate assessment of original products. Besides, further research can verify the role of usefulness in creativity evaluation. Diedrich et al. (2015) found that usefulness was the second criterion of creativity evaluation, which means usefulness can predict overall creativity when products have high novelty. This study also shows the difference in cognitive influences on usefulness and novelty, which also needs further researches. Besides, we conducted the experiment on the internet, which meant the experiment environment was limited, and the sample size also needs to be adjusted in a more strictly controlled experiment environment.

## Data Availability Statement

The original contributions presented in the study are included in the article/supplementary material, further inquiries can be directed to the corresponding authors.

## Ethics Statement

The studies involving human participants were reviewed and approved by Institutional Review Board of Psychology of the Nankai University. The patients/participants provided their written informed consent to participate in this study.

## Author Contributions

CZ, Y-XC, Y-FL, Y-XH, and D-QL designed this study and collected and analyzed the data with the assistance of XZ. CZ, Y-XC, and XZ wrote the manuscript. XZ and HZ provided critical emendations. All authors contributed to the article and approved the submitted version.

## Conflict of Interest

The authors declare that the research was conducted in the absence of any commercial or financial relationships that could be construed as a potential conflict of interest.

## Publisher’s Note

All claims expressed in this article are solely those of the authors and do not necessarily represent those of their affiliated organizations, or those of the publisher, the editors and the reviewers. Any product that may be evaluated in this article, or claim that may be made by its manufacturer, is not guaranteed or endorsed by the publisher.
